# *De Novo*, Divergence, and Mixed Origin Contribute to the Emergence of Orphan Genes in *Pristionchus* Nematodes

**DOI:** 10.1534/g3.119.400326

**Published:** 2019-05-14

**Authors:** Neel Prabh, Christian Rödelsperger

**Affiliations:** * Department of Integrative Evolutionary Biology, Max-Planck-Institute for Developmental Biology, Max-Planck-Ring 9, 72076 Tübingen, Germany; † Department of Evolutionary Genetics, Max-Planck-Institute for Evolutionary Biology, August Thienemann Str. 2, 24306 Plön, Germany

**Keywords:** Orphan genes, *de novo* genes, taxonomically-restricted, frame-shift, annotation artifacts

## Abstract

Homology is a fundamental concept in comparative biology. It is extensively used at the sequence level to make phylogenetic hypotheses and functional inferences. Nonetheless, the majority of eukaryotic genomes contain large numbers of orphan genes lacking homologs in other taxa. Generally, the fraction of orphan genes is higher in genomically undersampled clades, and in the absence of closely related genomes any hypothesis about their origin and evolution remains untestable. Previously, we sequenced ten genomes with an underlying ladder-like phylogeny to establish a phylogenomic framework for studying genome evolution in diplogastrid nematodes. Here, we use this deeply sampled data set to understand the processes that generate orphan genes in our focal species *Pristionchus pacificus*. Based on phylostratigraphic analysis and additional bioinformatic filters, we obtained 29 high-confidence candidate genes for which mechanisms of orphan origin were proposed based on manual inspection. This revealed diverse mechanisms including annotation artifacts, chimeric origin, alternative reading frame usage, and gene splitting with subsequent gain of *de novo* exons. In addition, we present two cases of complete *de novo* origination from non-coding regions, which represents one of the first reports of *de novo* genes in nematodes. Thus, we conclude that *de novo* emergence, divergence, and mixed mechanisms contribute to novel gene formation in *Pristionchus* nematodes.

The sequencing of hundreds of genomes lead to the discovery of new genes that do not share protein sequence homology with previously known genes. Over the years, these genes have been referred as young, pioneer, or orphan genes (Dujon 1996; Khalturin *et al.* 2009; Tautz and Domazet-Lošo 2011). Orphan genes makeup a considerable fraction of every sequenced metazoan genome and as a result, the total number of orphan genes has far surpassed that of the known gene families (Khalturin *et al.* 2009; Tautz and Domazet-Lošo 2011). Recent studies have shown that the proportion of orphan genes tends to be higher in secluded species that are devoid of genome data from closely related lineages (Khalturin *et al.* 2009; Tautz and Domazet-Lošo 2011). Thus, deep taxon sampling of closely related species is needed to study their age, origin, and mode of evolution (Palmieri *et al.* 2014; Prabh *et al.* 2018; Stein *et al.* 2018).

The nematode *Pristionchus pacificus* is an established model organism, which has been used for comparative studies with *Caenorhabditis elegans* (Sommer and Sternberg 1996; Sommer 2015). Orphan genes constitute roughly one third of all *P. pacificus* genes (Borchert *et al.* 2010; Baskaran *et al.* 2015). Given that the estimated divergence time between *P. pacificus* and *C. elegans* is 75 (±15) mya (Prabh *et al.* 2018; Werner *et al.* 2018), hence the high fraction of orphan genes in *P. pacificus* can be attributed to the depleted taxon sampling around it (Prabh and Rödelsperger 2016). Thus, to overcome the limitation of this scarce taxonomic representation, we recently sequenced ten closely related nematode genomes (Figure 1A) (Rödelsperger *et al.* 2014, 2017; Prabh *et al.* 2018), which created a ladder-like phylogeny around *P. pacificus*. All ten genomes were generated within a single laboratory and were annotated using the same pipeline to minimize technical variations and maximize comparability (Prabh *et al.* 2018). The ensuing analysis was based on orthologous clustering and assignment of the resulting gene families into age classes. This revealed that younger age classes tend to be located on chromosome arms, show less evidence of expression, evolve more rapidly, and have a higher propensity of being lost (Prabh *et al.* 2018). However, in our previous work, we neither segregated gene families into orphan and conserved classes nor did we infer their mechanism of origin. Although several mechanisms for the emergence of orphan genes have been suggested, rapid divergence and *de novo* emergence remain the most widely accepted (Khalturin *et al.* 2009; Tautz and Domazet-Lošo 2011). Previously, the *de novo* emergence of an open reading frame from an ancestrally non-coding region had been considered highly unlikely (Jacob 1977). However, the initial finding of the first instances of *de novo* genes in *Drosophila*, yeast, *E. coli*, humans, and plants (Levine *et al.* 2006; Delaye *et al.* 2008; Cai *et al.* 2008; Heinen *et al.* 2009; Knowles and McLysaght 2009), inspired several subsequent efforts to identify and characterize *de novo* genes in many other organisms including mammals, insects, and viruses (Li *et al.* 2010; Wu *et al.* 2011; Carvunis *et al.* 2012; Xie *et al.* 2012; Murphy and McLysaght 2012; Sabath *et al.* 2012; Wissler *et al.* 2013; Chen *et al.* 2015; Ruiz-Orera *et al.* 2018; Vakirlis *et al.* 2018; Klasberg *et al.* 2018; Schmitz *et al.* 2018). While *de novo* gene origin is generally inferred through identification of an ancestrally homologous non-coding sequence in a closely related genome (McLysaght and Hurst 2016), recognition of gene birth through divergence is complicated by the heterogeneous mechanisms with various degrees of sequence change that make automated homology detection untenable (Schmid and Tautz 1997; Schmid and Aquadro 2001; Long *et al.* 2003; Chen *et al.* 2013). Thus, investigating whether an orphan gene fits one of these models is a difficult proposition and requires both exhaustive computational and manual analysis of individual cases. Accordingly, this study is divided in two parts. In the first part, we employ an automated pipeline that establishes distinct classes of orphan genes and takes stock of how these genes are distributed along the *Pristionchus* phylogeny. In the second part, we manually investigate a limited number of candidate genes to illustrate several mechanisms of orphan gene origin.

**Figure 1:**
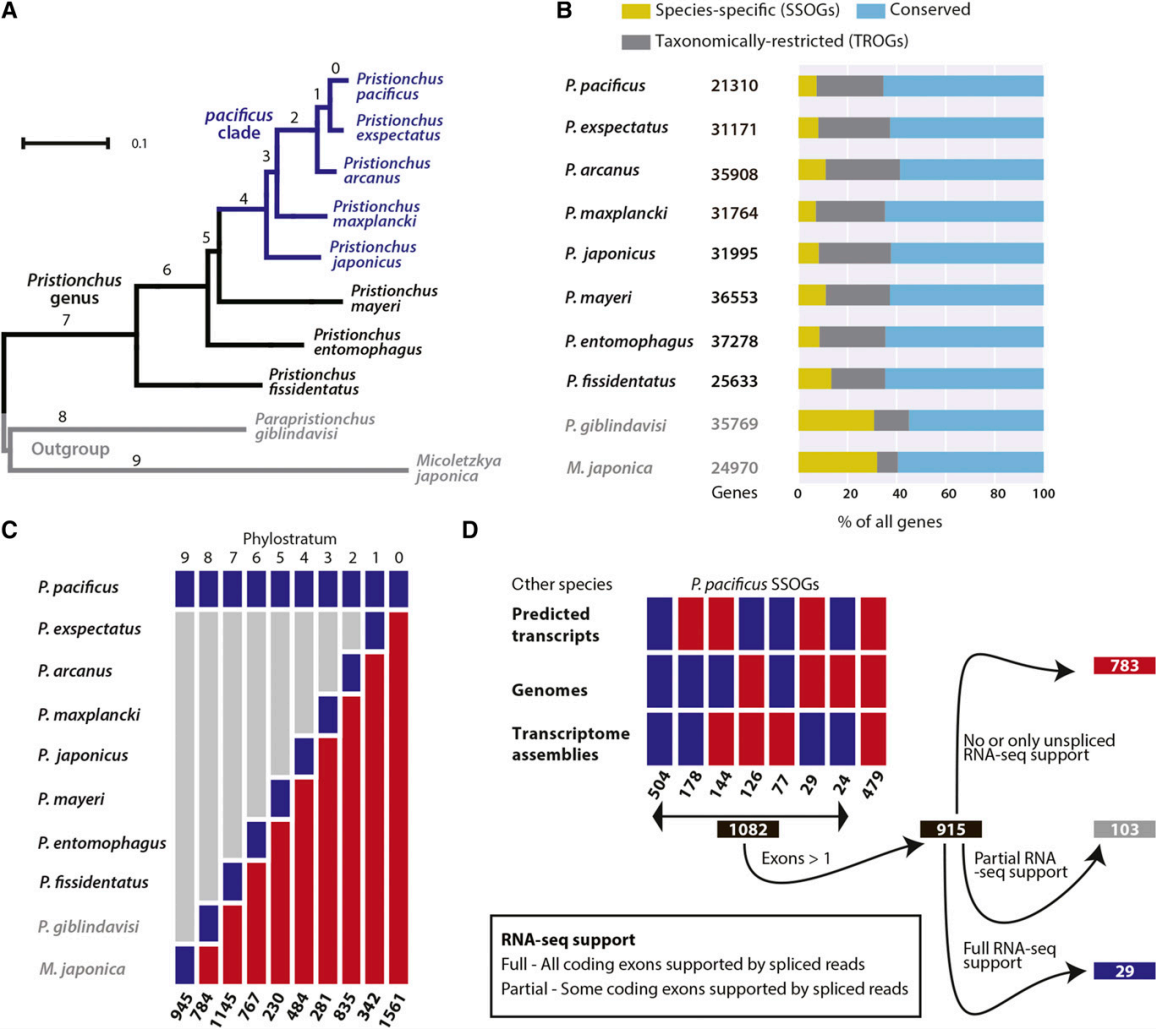
Fraction of SSOGs is consistent within the *Pristionchus* genus irrespective of divergence time (A) The maximum-likelihood phylogenetic tree of the species analyzed in this study, adapted from Rödelsperger *et al.* (2018). Branch lengths denote the number of amino acid substitutions per site. The numbers correspond to the phylostrata from panel C. (B) The horizontal stacked bars show the fractions of Conserved genes, TROGs, and SSOGs. (C) The ten phylostrata depict the origin of *P. pacificus* orphan genes along the diplogasrid lineage. Blue boxes indicate presence of *P. pacificus* orphan genes and the most distant diplogastrid species that has homologs of these gene, red bars indicate absence of homologs, and gray bars indicated homologs may or may not be present. The number of *P. pacificus* orphan genes in each phylostratum are at the bottom. (D) The heatmap shows traces of homology for *P. pacificus* in genomic and transcriptomic data of other species. The rectangles indicate whether traces of homology were found (blue) or not (red). Manual inspection of *P. pacificus* RNA-seq data resulted in a high-confidence data set of 29 *P. pacificus* SSOGs which were taken as the starting point for origin analysis.

## Methods

### Identification of orphan genes

The genome, protein and transcript data of 24 non-diplogastrid nematodes were obtained from Wormbase (WormBase web site, http://www.wormbase.org, release WS254, date 7/18/16). The phylogenomic data set for the ten diplogastrid nematodes was gathered from our previous publication (Prabh *et al.* 2018) and is available at http://www.pristionchus.org/download. To improve readability, we have abbreviated the original gene identifiers throughout the manuscript and a table with full identifiers and corresponding gene models on WormBase (WS269) and WormBase ParaSite (WBPS13) is provided in Table S2. All the Uniprot knowledgebase taxonomic divisions SwissProt data were downloaded from ftp://ftp.uniprot.org/pub/databases/uniprot/current_release/knowledgebase/taxonomic_divisions/. The invertebrate taxon contained a single *Pristionchus* species gene, Q9NHZ4, which was removed from further analysis.

We first identified all conserved genes for the ten diplogastrid nematodes using the following approach:

1. Classify all genes that have blastp match (E-value ≤ 10^−3^) with any non-diplogastrid nematode protein as ‘Conserved genes’. For the remaining genes go to step 2.
2. Classify all genes that have a tblastn match (E-value ≤ 10^−5^) with any non-diplogastrid nematode genome as ‘Conserved genes’. For the remaining genes go to step 3.
3. Classify all genes that have blastp match (E ≤ 10^−3^) with any protein from any Uniprot knowledgebase taxonomic divisions as ‘Conserved genes’. The proteins classified as conserved genes at this step are candidates for horizontal gene transfer.

The remaining genes were classified as ‘Orphan genes’. All blast runs were conducted, with version 2.6.0+, under default parameters (including no filtering of low complexity regions by SEG) unless mentioned otherwise.

### Classification of orphan genes

The availability of ten diplogastrid genomes provided us with the opportunity to further investigate *Pristionchus* orphan genes. Our first aim was to identify the orphan genes that have a homolog in at least one other diplogastrid species. Thus, for each species we selected the subset of orphan genes that have blastp match (E-value ≤ 10^−3^) with at least one other diplogastrid species. This subset of orphan genes was classified as ‘Taxon-restricted orphan genes’ (TROGs). The remaining orphan genes were classified as ‘Species-specific orphan gene’ (SSOGs), as they did not show blastp match with any other species. It is important to note here that for the identification of TROGs we have only used protein homology. We did not employ tblastn against genomes to avoid detection of pseudogenes or non-coding genomic regions as protein homologs. Further, since a ladder-like species phylogeny exists around our focal species *P. pacificus* (Figure 1A) (Rödelsperger *et al.* 2018), we decided to trace the origin of *P. pacificus* TROGs and SSOGs on this phylogeny. For this, we employed the phylostratigraphy approach (Domazet-Loso *et al.* 2007). This approach is based on finding the oldest ancestral node of a given phylogenetic tree where the founding member of a gene family can be traced back to. Thus, we divided the diplogastrid family tree into nine phylostrata. ‘Phylostratum 1’ corresponds to the most recent common ancestor of *P. pacificus* and *P. exspectatus*. Additionally, we created ‘Phylostratum 0’ that includes *P. pacificus* SSOGs and hence is the youngest phylostratum.

### Mapping of gene models from on the genome of other species

The synteny relation between genes from *P. pacificus* and the other species was derived using CYNENATOR (Rödelsperger and Dieterich 2010). Pairwise blastp results for each species pair and two files containing genomic location of genes in both the species, were provided as input to the software. The output file contained a list of genes from both species within the syntenic blocks. Spliced alignment of gene models from one species to the genome of another species was done by employing the protein2genome model of the Exonerate tool (Slater and Birney 2005).

### Gene structure validation

One of the main aims of this study was to elucidate the mechanistic details underpinning the birth of new genes. However, even with our structured approach of dividing the orphan genes into several categories and subcategories, we were unable to put forward a clear hypothesis on this matter. Thus, we decided to create a set of most reliable candidate genes to better understand the processes that foster new *P. pacificus* genes. For this, we limited ourselves to the *P. pacificus* SSOGs with confirmed gene structure. The validation of predicted gene structure was done by visual inspection, in IGV (Thorvaldsdóttir *et al.* 2013), of raw RNA-seq data aligned with the *P. pacificus* genome (Sinha *et al.* 2014; Prabh *et al.* 2018). We used TopHat v2.1.1 and STAR version 020201 for aligning the raw reads to genome (Dobin *et al.* 2013; Kim *et al.* 2013). Single exon genes were filtered out. Only multi-exon genes with minimum two spliced RNA-seq reads aligning all coding exons and minimum two spliced reads straddling such exons, were assigned ‘fully confirmed gene structure’ status. If, only few, but not all exons of a gene qualified this criteria, then it was assigned ‘partially confirmed gene structure’ status. For overlapping genes from opposite strands, strandedness of strand-specific RNA-seq data were used as an additional confirmation step.

### Selection analysis

For selection analysis of the SSOG candidates, their orthologous reading frames (including in-frame stop codons) from sister species were extracted and manually adjusted. Protein alignment of the candidate and its corresponding reading frames from one or more sister species was done using MUSCLE and visualization was done with SeaView (Edgar 2004; Gouy *et al.* 2009). The protein alignment was converted to codon with PAL2NAL (Suyama *et al.* 2006). Selection analysis was done with codeml suite of PAML (Yang 2007). Species tree was passed as gene tree to PAML. If the corresponding homologous region from only one sister species was included in the analysis we generated a single ω value for the entire tree, else we generated independent ω values for each branch of the tree (Figure 5C). The statistical significance of the resulting ω values was calculated using the likelihood ratio test at the P-value threshold of 0.05. Only statistically significant results were reported.

### Data availability

Sequences are available at http://www.wormbase.org and http://www.pristionchus.org. Full accession numbers of all abbreviated gene identifiers are listed in Table S2. Supplemental material available at FigShare: https://doi.org/10.25387/g3.8123768.

## Results

### Roughly 10% of all genes are species-specific irrespective of sampling depth

To quantify the amount of orphan genes among the ten nematode genomes, we applied a three-step filtering procedure (see *Methods*) that classified around one third of genes in each genome as orphan (Figure 1A). We next explored the conservation of orphan genes within the diplogastrida family. Roughly 70% of all orphan genes have a homolog in at least one other diplogastrid species (Figure 1B and Figure S1) and were therefore labeled as ‘Taxonomically-restricted orphan gene’ or ‘TROG’. Thus, approximately 10% of all genes in different *Pristionchus* species lack any homology at the protein level with any other species and were classified as ‘Species-specific orphan gene’ (SSOG). This lack of phylogenetic signal is unexpected, since the taxonomic sampling is much deeper around our focal species *P. pacificus* (Figure 1A) and encompasses the two sister species, *P. exspectatus* and *P. arcanus*, that can still form viable but sterile hybrids with *P. pacificus* (Kanzaki *et al.* 2012). Hence we naively anticipated that this should result in a much lower fraction of SSOGs in our focal species and its close neighbors. While we cannot rule out that a constant fraction of erroneous gene annotations partially contributes to this pattern, these results are consistent with the idea that novel genes are frequently generated as a result of pervasive transcription but rarely reach fixation and are rapidly lost (Schmitz *et al.* 2018).

### SSOGs make the most gene rich phylostratum

To gain more detailed insights into the age distribution of *P. pacificus* orphan genes, we separated them into different phylostrata that can be mapped to the most recent common ancestors of *P. pacificus* and the other diplogastrid species (Figure 1C). Based on the parsimonious assumption that the breadth of a gene’s phylogenetic distribution is an indicator of its age, a gene that is shared by several species is expected to be older than a gene that is present in only one or two species. Thus, each orphan gene was placed into the phylostratum that points to the most recent common ancestor of *P. pacificus* and its most distantly related species that has a homolog of this gene (Domazet-Loso *et al.* 2007). *P. pacificus* SSOGs were placed in the ‘Phylostratum 0’ which is the most gene rich among all phylostrata (Figure 1C). This gene set is likely a mixture of annotation artifacts and novel gene-like sequences that result from pervasive transcription and translation but do not live long enough to survive a speciation event (Hangauer *et al.* 2013; Ruiz-Orera *et al.* 2018; Schmitz *et al.* 2018). Due to the high abundance of *P. pacificus* SSOGs and the possibility to study their origin in multiple closely related genomes, we decided to investigate in further detail the processes that generate such SSOGs.

### Most P. pacificus SSOGs have traces of homology in closely related genomes

The taxon sampling around our focal species *P. pacificus* allowed exhaustive homology search of *P. pacificus* SSOGs in the genomes of sister species, which could be indicative of their mechanism of origin. To this end, we performed various blast searches against the annotated transcripts, genome assembly, and transcriptome assembly (Figure 1D). While tblastn searches against the genome assembly of other species may identify homologous non-coding regions of *de novo* candidates, we additionally performed a blastn search against the annotated transcripts to screen for potential cases of ORF switching, and a blastn search against the transcriptome assembly to assess the degree of missing homology due to assembly gaps. As a result, 504 (32%) of *P. pacificus* SSOGs show blast hits in all three target database types, which after closer investigation was seen to be largely due to overlapping gene structures. It is important to note here that nematodes possess high fraction of overlapping genes (Jan *et al.* 2010; Rödelsperger *et al.* 2016). Another 479 (31%) of *P. pacificus* SSOGs did not show hits in any of the databases and were labeled ‘Untraceable’. Among the remaining SSOGs, we find only 29 (2%) with a hit in the transcriptome assembly but not in the genome or the annotated transcripts. This fraction of putative assembly gap genes is constantly low for all our genomes supporting their comparably high quality (Prabh *et al.* 2018). In total, 1082 (61%) of *P. pacificus* SSOGs exhibit detectable traces of homology in the genomes of other closely related species, demonstrating that the taxon sampling of our phylogenomic data set is sufficient to study the mechanisms of origin for the most *P. pacificus* SSOGs in greater detail.

### Identification of a high-confidence candidate set for origin analysis

Given that more than a thousand *P. pacificus* SSOGs show traces of homology in closely related sister species and that the gene structures of orphan genes in general are poorly supported by expression evidence (Prabh and Rödelsperger 2016), we first needed to define a high-confidence candidate set of SSOGs that could be used for detailed gene origin analysis (Figure 1D). We only considered SSOGs with more than one annotated exon, because we hypothesized that this additional layer of regulated expression involving the proper splicing of the transcripts would yield a more likely protein-coding gene candidate with confirmed regulated expression as opposed to pervasive transcription and translation (Hangauer *et al.* 2013; Ruiz-Orera and Mar Albà 2019). Additionally, the splice sites can be informative to better predict the correct orientation of the gene, which is essential to elucidate their origin and the reason why homology was not detected in the first place. We manually inspected RNA-seq alignments of all *P. pacificus* SSOGs except the untraceable genes, in total 1082 candidate loci, to find gene structures that are fully confirmed by raw RNA-seq reads and we insisted on finding a minimum of two raw RNA-seq reads aligned with each coding exon and two spliced reads that span such exons. Eventually, we established 29 SSOGs with fully confirmed gene structures (Figure 1D) that formed our high-confidence candidate set. Based on our investigation, we provide examples for six plausible mechanisms that explain the origin of SSOGs including two examples of *de novo* genes. Among the high-confidence candidates 21 can be explained by the proposed mechanisms, the origin of six candidates cannot be unambiguously concluded and the two remaining candidates were annotation artifacts (Table S1).

### Divergence by recycling of ancestrally protein-coding fragments

**Figure 2:**
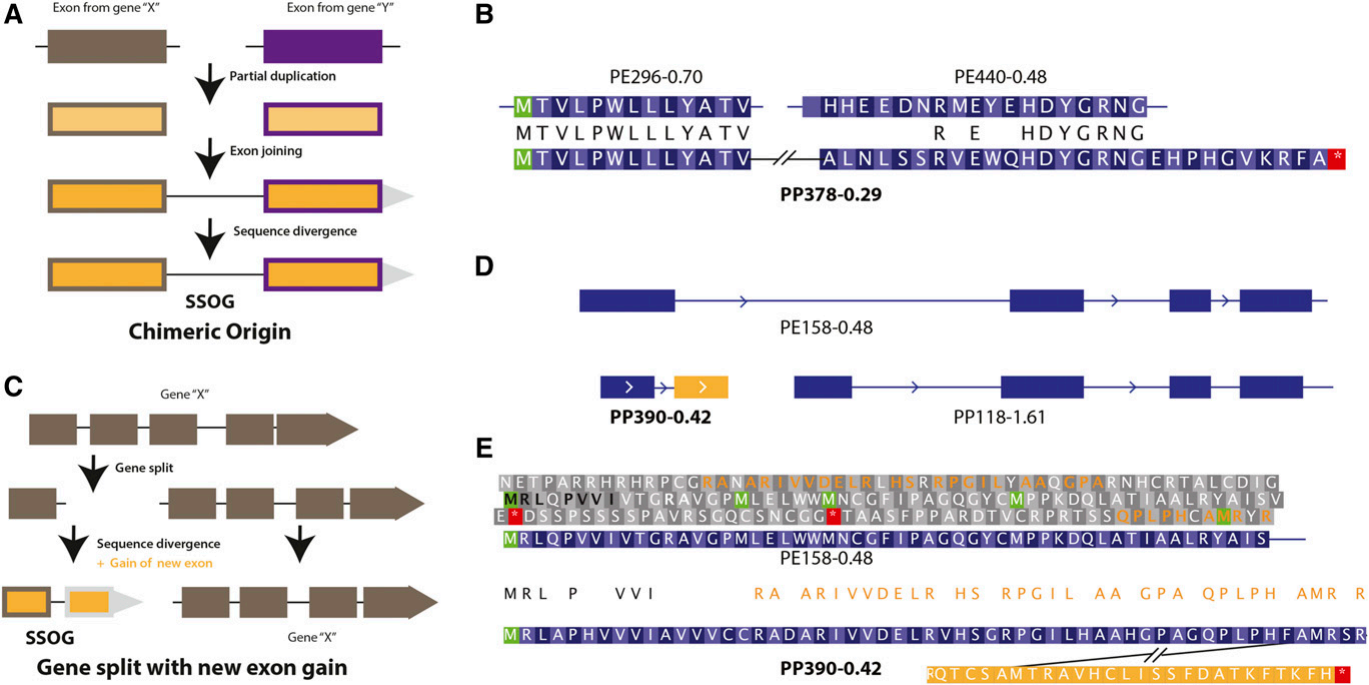
Sequence divergence and ORF shift erode evidence of homology. (A) The schematic overview shows an example of an SSOG with chimeric origin. Two exons gained from partial duplication of two distinct genes are joined together and with time sequence divergence occurs. Thus, traces of sequence homology with the original exons become hard to detect and such genes get classified as SSOGs. (B) This example shows a *P. pacificus* SSOG (PP378-0.29) of chimeric origin and its alignments with parts of two conserved *P. exspectatus* genes. Identical amino acid residues are labeled in black between the *P. pacificus* and *P. exspectatus* exons. Even though the first exon is 100% identical with its homolog, the stretch of alignment is not long enough to be detected by blastp at the stipulated E-value cutoff. (C) Schematic overview of a gene split with subsequent exon gain which results in an SSOG (D) The *P. pacificus* SSOG PP390-0.42 is homologous to the first exon of a conserved *P. exspectatus* gene. The neighboring gene shows homology with the remaining exons, indicating that the SSOG is derived from a gene split event. (E) The alignment of the *P. pacificus* SSOG with *P. exspectatus* is spread over multiple reading frames. Amino acid identity between the predicted reading frame of both the proteins are marked in black and those from the other reading frame of the exspectatus gene are marked in saffron. The residues corresponding to the *P. pacificus* SSOG in different reading frames of the *P. exspectatus* sequence are also labeled in black.

The first mechanism alludes to chimeric gene formation resulting in an SSOG with two exons. Both exons are derived by partial duplication, but of separate source genes. The paralogous exons from both the ancestral source genes are duplicated and then get inserted in close proximity to facilitate the formation of a novel ORF (Figure 2A). Considering that such genes can be created by minimal contribution from existing genes, local alignment based tools may fail to detect the homology of these short sequence stretches with their paralogous exons. For example, PP378-0.29 is a *P. pacificus* SSOG with two exons. Its first exon has 100% protein identity with an exon from a *P. exspectatus* Gluthatione peroxidase gene (92% identity with the corresponding exon of the orthologous *P. arcanus* gene PA7-2.29), while its second exon shows partial identity with an exon of another conserved *P. exspectatus* gene (PE440-0.48, Figure 2B). Orthologs of both *P. exspectatus* genes are maintained in *P. pacificus* and given that the first exon shows high sequence identity with same exon of the Gluthatione peroxidase gene in both *P. exspectatus* and *P. arcanus*, we can establish that the the first exon of our candidate has been derived through partial duplication of an existing gene. However, blastp failed to detect homology with the paralogous exons from the two *P. exspectatus* genes. This demonstrates that even if a high percentage of identity is retained between paralogous exons, small chimeric genes can be classified as SSOGs.

The second mechanism of SSOG creation is based on splitting of an ancestral gene (Figure 2C). After the split, either both or one of the fragments can diverge from the ancestral sequence and can also acquire new exons. If the fragments resulting from a gene split event are small, a moderate level of divergence can result in a failure to detect homologous sequences. The *P. pacificus* gene PP390-0.42, is an example of an SSOG created by gene split (Figure 2D). Based on synteny information and spliced alignment, we mapped the first exon of this gene to the first exon of a conserved gene (PE158-0.48) in *P. exspectatus* and another *P. pacificus* gene is homologous to the remaining exons of the *P. exspectatus* gene. The *P. exspectatus* gene PE158-0.48 is the ortholog of *P. arcanus* gene PA73.-2.42 and both genes share the same first exon, which confirms that the first exon of our candidate gene is the result of a gene split event. Upon manual inspection, we found that the first exon of the *P. pacificus* SSOG has acquired insertions that shifted its reading frame and renders protein homology undetectable. Although some of the N-terminal residues are identical to the *P. exspectatus* protein (Figure 2e), the remaining residues from the first exon of our candidate gene were found to be derived from other reading frames of the orthologous *P. exspectatus* exon. Hence, it is clear that the predicted ORF from the first exon of our candidate gene is mainly derived from the non-ancestral reading frame. Moreover, the initial segment, which partially retains the ancestral ORF, is not large enough to facilitate homology detection. Ancestry of the second exon of the *P. pacificus* SSOG could not be established even after manual inspection. This suggests that the second exon has been acquired *de novo*. Thus, origin of the candidate gene can be attributed to gene split, partial ORF shift, and *de novo* acquisition of a new exon.

### New gene creation through alternative reading frame usage

**Figure 3:**
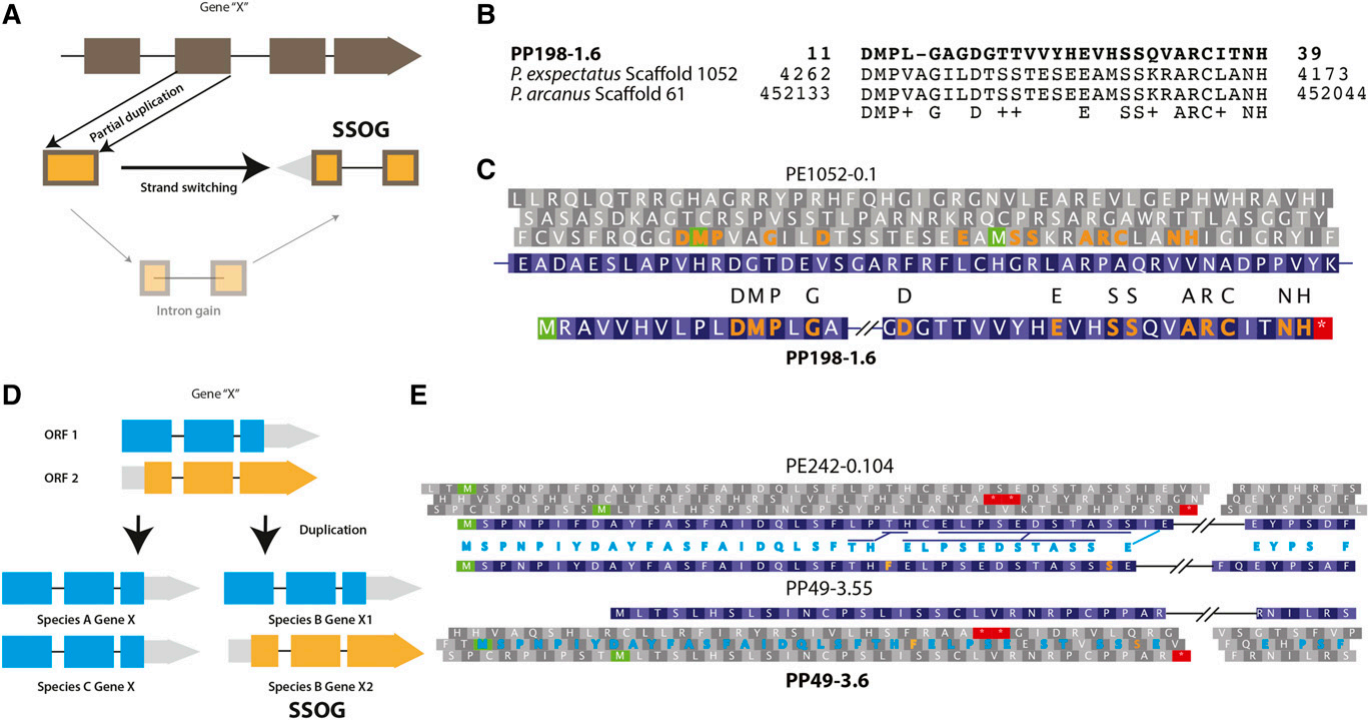
Switching to an alternate reading frame gives rise to SSOGs. (A) Partial duplication in combination with an intron gain can allow opening of a new reading frame from the opposite strand. (B) *The P. pacificus* SSOG, PP198-1.6, is an example of both strand switching and exon splitting. Here we show amino acid identity and similarity between our candidate SSOG with the translation from *P. exspectatus* and *P. arcanus* genomes. (C) This is a two exon gene, and both the exons share a remote homology with the opposite strand of one single exon of a *P. exspectatus* gene at the aligned locus. The identical amino acid residues between the *P. pacificus* SSOG and its corresponding *P. exspectatus* ORF are marked in saffron. (D) The schematic overview illustrates a case of actualisation of an alternative reading frame by duplication. Overprinting describes a gene with two alternate ORFs. Gene prediction tools generally do not annotate alternate overlapping ORFs from the same strand. However, duplication might generate gene copies where the alternative ORF will be annotated. Nevertheless, in species with a single copy of this gene only one ORF gets predicted and due to lack of protein homolog in other species the alternate ORF will be categorized as SSOG. (E) PP49-3.6 is a four exon SSOG. Its *P. exspectatus* homolog is predicted from the same strand but in a different reading frame. Both genes maintain both ORFs. We found a *P. pacificus* gene, PP49-3.55, which is predicted in the *P. exspectatus* ORF and their identical amino acid residues are marked in turquoise between their exons and also in corresponding reading frame of our candidate SSOG. Comparison of this reading frame between the two *P. pacificus* genes shows two residues, in saffron, that are uniquely found in these genes. This indicates that SSOGs can be generated by prediction of an alternate ORF.

So far, we have discussed two mechanisms of new gene creation that require deviation from an existing gene structure but maintain the ancestral reading frame either fully or partially. Here we discuss a third mechanism that involves strand switching, which results in a completely new ORF (Figure 3A). The *P*. *pacificus* SSOG PP198-1.6 has two coding exons and is an example of such a mechanism. In *P. pacificus*, this gene is placed within an intron of a conserved *P. pacificus* gene (Figure S2). This intron is 2.1 kb long in *P. pacificus*. The corresponding intron of the *P. exspectatus* ortholog is 1.4 kb long and shows no homology to our candidate SSOG at the nucleotide or the protein level (Figure S2). Spliced alignment of the candidate SSOG on to the *P. exspectatus* genome did not generate any match. Thus, we performed a tblastn match against both the *P. exspectatus* and *P. arcanus* genomes at a relaxed threshold of E-value < 10 (Figure 3B). The resulting aligned genomic section was traced to a single exon of PE1052-0.1 gene whereby our candidate has some sequence identity with a reading frame from the reverse strand of the *P. exspectatus* gene (Figure 3, B and C). The ortholog of PE1052-0.1 gene in *P. arcanus* (PA61-4.37) also maintains this exon and the neighboring exon-intron boundaries. Although the sequence identity between PP198-1.6 and PE1052-0.1 is not substantial (tblastn E-value = 2.37) and could be indicative of an ancient duplication event with subsequent losses, we propose that our candidate SSOG gene shares a common ancestry with sequences in *P. exspectatus* (PE1052-0.1) and *P. arcanus* (PA61-4.37) and originated from a combination of a possibly ancient duplication event, intron gain, strand-switching (Figure S2), and insertion at its current position.

The fourth mechanism deals with genes that can have more than one overlapping ORFs. This phenomenon is known as overprinting and has been reported in several studies (Grassé 1977; Ohno 1984; Keese and Gibbs 1992; Chen *et al.* 1997; Makalowska *et al.* 2005; Nekrutenko *et al.* 2005; Chung *et al.* 2007; Gontijo *et al.* 2011; Sabath *et al.* 2012; Guan *et al.* 2018). Generally, gene prediction tools only annotate single ORFs. However, if an ancestral gene with two ORFs gets duplicated in a lineage, one of the duplicates can switch to the less common ORF (Figure 3D). This will lead to classification of the duplicated gene as an SSOG, as the corresponding ORF has not been annotated in any other species. We found that the *P. pacificus* SSOG PP49-3.6 is one candidate for such a scenario. Although it lacks protein homologs with any other species, this gene has a paralog, PP49-3.55, at the predicted transcript level (blastn E-value = 0.00, identity = 92%). The protein predicted from the candidate SSOG is in a reading frame that differs from that of its paralogous transcript. We found that both ORFs are available to both paralogs. The predicted ORF of the paralog is conserved within the genus and has its orthologous ORF in *P. exspectatus* (Figure 3e). Selection analysis indicates that the predicted *P. pacificus* ORF shows an ⍵ value of 1.6 whereas the ancestral ORF shows evidence of negative selection (⍵ = 0.38). This demonstrates how annotation artifacts such as inconsistent ORF calling can give rise to classification of genes into SSOGs. However, in the absence of conclusive evidence such as ribosome profiling data, we cannot completely reject the predicted reading frame and would point out the possibility that gene duplication in principle allows actualisation of such alternative ORFs.

### Heuristic failures in homology detection contribute to classification as SSOGs

**Figure 4:**
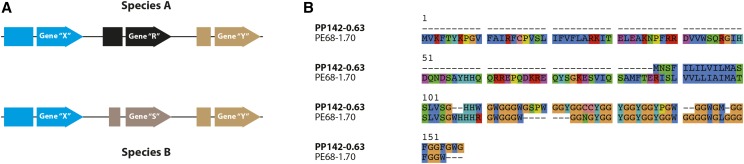
Failures in homology detection lead to classification as SSOGs. (A) Conserved synteny may reveal loci where genes are incorrectly classified as SSOGs due to a failure of homology detection. (B) The *P. pacificus* SSOG PP142-0.63 is found in a conserved syntenic region with the *P. exspectatus* TROG PE68-1.70. Both proteins are ‘GGX’ repeat rich proteins and share a small non-repetitive part, but blastp failed to identify both proteins as homologous.

The fifth mechanism of SSOG formation specifically deals with the fact that blast programs implement a heuristic approach to find sequence matches and typically these programs are run with default settings. It is obvious that lowering thresholds (*e.g.*, E-value) or switching to a more sensitive alignment approach (Slater and Birney 2005) facilitates the identification of homologous sequences for a number of *P. pacificus* SSOGs that were missed by blast programs. This has been illustrated by the identification of homologous regions for the previously described divergence cases (Figure 2B and Figure 3B). During our investigation of high-confidence candidates, we encountered two repeat rich SSOGs, PP142-0.63 and PP81-0.14, where more detailed investigation of the syntenic region facilitated the identification of a homologous segment in the *P. exspectatus* genome (Figure 4A). Even when blast’s repeat filtering is switched off, it fails to detect homology due to the combination of a small non-repetitive match and indels as well as substitutions in the repeat-rich region (Figure 4B). Even though we cannot be sure, how specific this behavior is to repeat-rich genes, these two examples together with the previous examples illustrate that the failure of any heuristic approach to detect homology, will inevitably lead to the classification of certain genes with homologs as SSOGs.

### Evidence for de novo genes in P. pacificus

**Figure 5:**
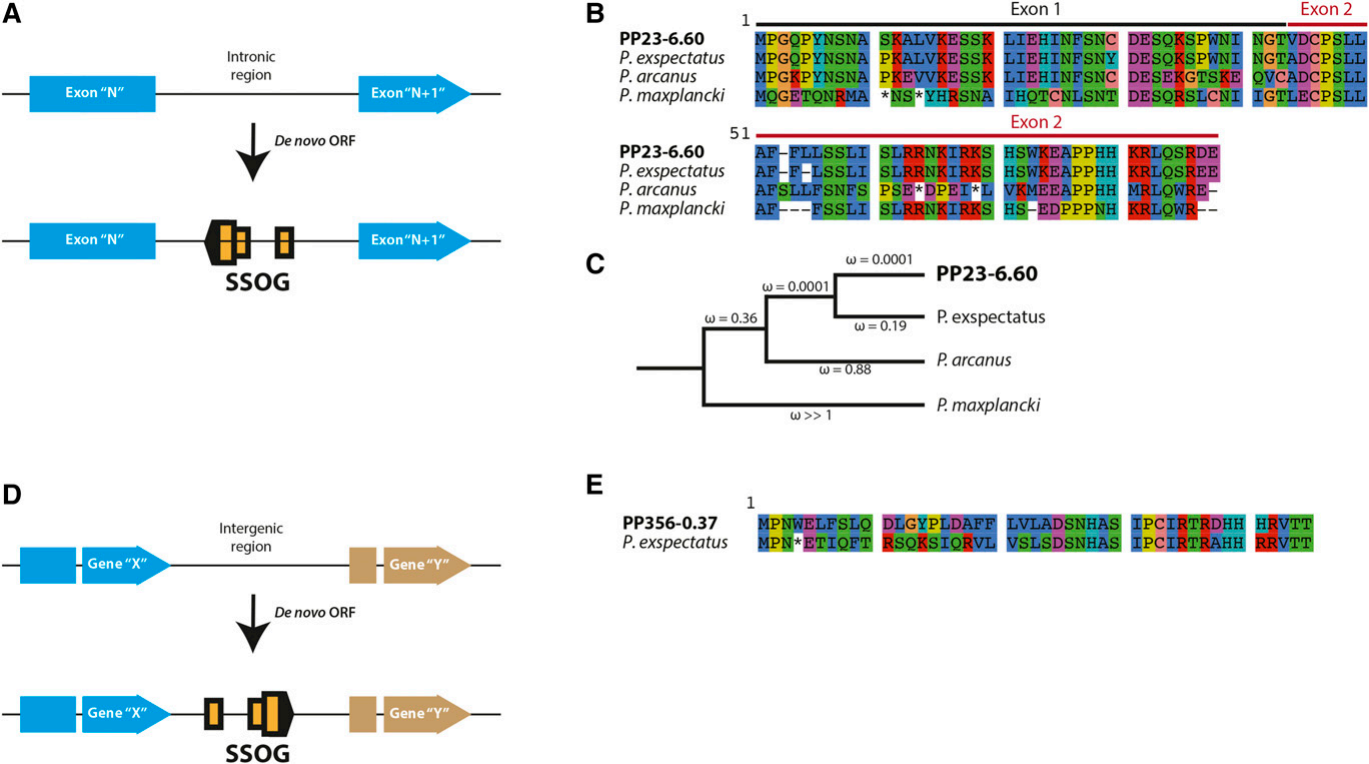
*De novo* gene birth. (A) A *de novo* gene can originate as an antisense transcript in the intron of another gene. *De novo* creation of such an ORF can be verified by finding the corresponding intron in a related species that lacks this ORF. (B) PP23-6.60 is two exon *P. pacificus* gene that is located in an intron of another *P. pacificus* host gene. Based on the identification of the orthologous intron of the host gene in other species, we have created an alignment of our candidate and translation of its corresponding reading frame from other species. It is clear that the same ORF also exists in *P. exspectatus*. However, *P. arcanus* has two stop codons (*) in the middle of the 2^nd^ exon and *P. maxplancki* has two stop codons in the 1^st^ exon itself. (C) Selection analysis done on the alignment from panel B, indicates that the predicted ORF has been under strong selection toward the *P. pacificus* lineage. This trend may have started from the common ancestor of *P. pacificus*, *P. exspectatus* and *P. arcanus*. (D) A *de novo* gene can originate from ancestrally intergenic region. (E) The *P. pacificus* gene PP356-0.37 contains a single coding exon and its homologous reading frame in *P. exspectatus* is found at a non-transcribed intergenic location and has an early stop codon (*). This gene does not show sequence homology with any other species but *P. exspectatus*.

All the five mechanisms described in the previous sections portray how new genes can be created from old genes. However, the *P. pacificus* SSOG PP23-6.60 is an example of *de novo* formation from an ancestrally non-coding region. It has two coding exons, placed within a single intron of the *P. pacificus* homolog of *C. elegans* C27F2.7 (Figure S3). The intronic location of our candidate SSOG within a conserved gene helped us to identify the orthologous genomic locations in other *Pristionchus* species. Based on the spliced alignment of our candidate against the genomes of other species we were able to extract the orthologous sequences from *P. exspectatus*, *P. arcanus* and *P. maxplancki* (Figure 5B). No transcriptional evidence for the genomic regions corresponding to their extracted ORFs was found in *P. exspectatus*, *P. arcanus* and *P. maxplancki* (Figure S3). Nevertheless, the length of the *P. exspectatus* ORF matches that of the *P. pacificus* prediction. Additionally, the *P. arcanus* ORF aligns well with the *P. pacificus* ORF but contains two stop codons in the middle of the second exon. Furthermore, the sequence extracted from *P. maxplancki* has stop codons at the 11^th^ and 14^th^ position and no Methionine thereafter to make an abridged ORF. This suggests that the ORF at this locus was engendered in the common ancestor of *P. pacificus*, *P. exspectatus*, and *P. arcanus*. Moreover, the lack of ORF in *P. maxplancki* and alignable region in other species confirms the *de novo* origin of this gene. The protein coding nature of our *de novo* candidate was further supported by selection analysis of the *P. pacificus* ORF and the protein translation from the other species. In this analysis, we allowed each branch of the tree to have an independent ω value. Here, the branches leading from the common ancestor of *P. pacificus*, *P. exspectatus* and *P. arcanus*, toward the *P. pacificus* lineage are under extremely strong negative selection (Figure 5C). This indicates that since its emergence, the *de novo* gene has been maintained as a protein coding gene in the lineage leading to *P. pacificus*.

Our second *de novo* candidate PP356-0.37 is a two exon gene with its entire coding sequence in the 2^nd^ exon. Since the candidate could be mapped on to the genomes of none of the other species but *P. exspectatus*, we were only able to extract the orthologous *P. exspectatus* sequence from a conserved syntenic region (Figure S4). Nevertheless, the absence of transcription in *P. exspectatus* and the presence of a stop codon at the 4^th^ position of the extracted *P. exspectatus* sequence confirms the non-genic and non-transcribed status of the *P. exspectatus* sequence. Even though the absence of homologous traces outside the two sister species did not allow us to conclusively infer the state of this gene in the ancestor of *P. pacificus* and *P. exspectatus*, we propose that the *P. pacificus* SSOG PP356-0.37 arose very recently and is a putative *de novo* gene that emerged from a previously non-coding intergenic region in the *P. pacificus* lineage. Together with a recent study of the *Caenorhabditis* genus (Zhang *et al.* 2019), these genes are the first examples of *de novo* genes in nematodes.

## Discussion

Genome sequencing projects identify novel genes in all domains of life. Many of these genes have been shown to be involved in lineage specific adaptations (Milde *et al.* 2009; Kawasaki *et al.* 2011; Mayer *et al.* 2015; Villanueva-Canas *et al.* 2016; Aguilera *et al.* 2017). However, even with deep taxonomic sampling of genomic data sets, it remains unclear, what are the most common mechanisms to form novel genes. Given, that yeasts, mammals, insects, and nematodes have highly variable genomic architectures (*e.g.*, genome size and fraction of coding sequences (Rödelsperger *et al.* 2013), presence of operons (Sinha *et al.* 2014), recombination (Srinivasan *et al.* 2002), transposon control (Sarkies *et al.* 2015), and DNA methylation (Rošić *et al.* 2018)), multiple studies in different clades are needed to characterize and compare processes that lead to emergence of novel genes. In this study, we bring the power of clade genomics to enumerate various mechanisms of gene birth in *Pristionchus* nematodes (Rogers 2018), this makes our study the first of its kind in nematodes.

The exceptionally high number of SSOGs (Figure 1, B and C) may be due to a combination of erroneous gene models and short-lived gene-like sequences that result from pervasive transcription and translation (Hangauer *et al.* 2013; Schmitz *et al.* 2018; Ruiz-Orera and Mar Albà 2019). We discussed two cases, where either wrong ORF annotation or heuristic failure in homology detection resulted in an incorrect classification as SSOGs. However, as most SSOGs are only poorly supported by expression data, it is challenging to conclusively distinguish annotation artifacts from lowly expressed genes. Thus, it remains unclear, to what extent annotation errors and pervasive transcription and translation contribute to the abundance of SSOGs. The deep taxon sampling of our phylogenomic data allowed us to detect traces of homology for 1082 (61%) of *P. pacificus* SSOGs. This demonstrates the potential of the *Pristionchus* system to study the mechanisms of gene birth. However, in contrast to many other recent studies in mammals (Ruiz-Orera *et al.* 2018; Schmitz *et al.* 2018), insects (Klasberg *et al.* 2018) and yeasts (Carvunis *et al.* 2012; Vakirlis *et al.* 2018), we did not aim only for the identification and characterization of *de novo* genes, but undertook an unbiased exploration of high-confidence candidates through manual investigation of gene structures and various sequence search methods (*i.e.*, blastn, tblastn, and spliced-mapping with exonerate) in our phylogenomic data set. In some cases, this lead to a reclassification of an SSOG as TROG, but more importantly this demonstrated that both divergence of existing genic segments and *de novo* creation of new genic elements contribute to orphan gene emergence. While *de novo* origin only requires the identification of an ancestrally non-coding sequence in a closely related species, the case of the *P. pacificus* SSOG PP390-0.42 (Figure 2, C, D and E) shows that the distinction between *de novo* and divergence can sometimes be difficult to discern. The formation of this gene results from several steps, which include splitting of the ancestral gene, sequence divergence, reading frame shift and *de novo* acquisition of a new exon. Thus, we argue that this gene should be considered a product of ‘mixed origin mechanism’, as both divergence and *de novo* origin mechanisms have contributed to its birth. Moreover, in order to quantify the contribution of different origin mechanisms, we first have to establish a comprehensive catalog with detailed descriptions of all possible mechanisms and then develop computational tools to reliably detect them. This knowledge can be used in future to perform more systematic screens, possibly also on older phylostrata, in order to get better estimates of the relative contribution of various mechanisms to novel gene emergence.
